# Automatic single-trial discrimination of mental arithmetic, mental singing and the no-control state from prefrontal activity: toward a three-state NIRS-BCI

**DOI:** 10.1186/1756-0500-5-141

**Published:** 2012-03-13

**Authors:** Sarah D Power, Azadeh Kushki, Tom Chau

**Affiliations:** 1 Bloorview Research Institute, Holland Bloorview Kids Rehabilitation Hospital, Toronto, Canada; 2 Institute of Biomaterials and Biomedical Engineering, University of Toronto, Toronto, Canada

## Abstract

**Background:**

Near-infrared spectroscopy (NIRS) is an optical imaging technology that has recently been investigated for use in a safe, non-invasive brain-computer interface (BCI) for individuals with severe motor impairments. To date, most NIRS-BCI studies have attempted to discriminate two mental states (e.g., a mental task and rest), which could potentially lead to a two-choice BCI system. In this study, we attempted to automatically differentiate three mental states - specifically, intentional activity due to 1) a mental arithmetic (MA) task and 2) a mental singing (MS) task, and 3) an unconstrained, "no-control (NC)" state - to investigate the feasibility of a three-choice system-paced NIRS-BCI.

**Results:**

Deploying a dual-wavelength frequency domain near-infrared spectrometer, we interrogated nine sites around the frontopolar locations while 7 able-bodied adults performed mental arithmetic and mental singing to answer multiple-choice questions within a system-paced paradigm. With a linear classifier trained on a ten-dimensional feature set, an overall classification accuracy of 56.2% was achieved for the MA vs. MS vs. NC classification problem and all individual participant accuracies significantly exceeded chance (i.e., 33%). However, as anticipated based on results of previous work, the three-class discrimination was unsuccessful for three participants due to the ineffectiveness of the mental singing task. Excluding these three participants increases the accuracy rate to 62.5%. Even without training, three of the remaining four participants achieved accuracies approaching 70%, the value often cited as being necessary for effective BCI communication.

**Conclusions:**

These results are encouraging and demonstrate the potential of a three-state system-paced NIRS-BCI with two intentional control states corresponding to mental arithmetic and mental singing.

## Background

Many individuals with severe and multiple motor disabilities cannot communicate through the conventional avenues of speech and gesture. Many such individuals may also lack sufficient motor control to operate common movement-based access devices (e.g.,mechanical switches, eye-trackers) [1]. Brain-computer interface (BCI) technologies are controlled through brain activity alone, and may provide these individuals with an alternative, movement-free means of access [2]. Near-infrared spectroscopy (NIRS) is an optical imaging technology that has been recently investigated as a safe, non-invasive brain response measurement technology for potential use in BCI applications [3-6]. NIRS can be used to assess functional activity in the cerebral cortex via measurement of the haemodynamic response (see [7] for description of fundamental principles). NIRS offers a number of advantages for BCI applications compared to the more frequently studied electroencephalography (EEG), such as insensitivity to electrophysiological artefacts (e.g., EMG, EOG) and, when monitoring regions not covered by hair (e.g. prefrontal cortex), much faster and easier sensor placement (e.g., no need for electrode gel [8]). Note that NIRS optode placement in areas covered by hair is much more difficult, and may take considerably longer to achieve adequate signal-to-noise ratio.

Generally, a user controls a BCI output by consciously eliciting distinct, reproducible patterns of activation in a particular brain region. This is usually done by performing different mental tasks, such as motor imagery [3,4], mental arithmetic [5,6,9-12], mental singing [5,6] and verbal tasks [10,11]. The system then detects and interprets these patterns of activity, and produces the appropriate command signal to control a connected external device (e.g., computer cursor) in the way the user intended.

For the most part, previous research has investigated the development of NIRS-BCIs operating under synchronous control paradigms. Under synchronous control, the system evaluates the user's brain activity for control (i.e., is vigilant) only during certain periods defined by the system, and users must exert intentional control over their brain activity (i.e., generate what we refer to as an "intentional control (IC) state") during each and every one of these "system-vigilant" periods. Though functional, the need for such frequent mental state control is mentally demanding for the user. An attractive alternative to the synchronous control paradigm is "system-paced" control, a paradigm proposed by Mason et al. [13], in which users are required to intentionally control their brain activity only during the system-vigilant periods in which they actually wish to affect the BCI output, and can remain in a more natural, "no-control (NC)" state at all other times. This paradigm can be considered an intermediate step between synchronous control and the "ideal" asynchronous, or self-paced, paradigm. See [14] for a full discussion of the different BCI control paradigms and their implications for NIRS-BCI.

In a previous study, we investigated the feasibility of a system-paced NIRS-BCI with one IC state corresponding to the performance of either mental arithmetic (MA) or mental singing (MS) [14]. These tasks were chosen as they had both been previously shown to elicit activation in the prefrontal cortex [5,6,9,10,12,15-19]. The precise conditions that induce prefrontal activation during mental arithmetic are not well understood [20], but could be associated with working memory [21,22], mental stress [23,24], or other general cognitive operations that are instrumental, but not specific, to mental arithmetic [21,25]. Music is known to elicit [26,27] intense emotional responses that activate brain regions believed to be associated with emotional behaviors, including the prefrontal cortex [28] and specifically, the orbitofrontal and frontopolar areas [29,30]. We found that both mental arithmetic and music imagery could be automatically distinguished from the NC state with average accuracies of 71.9% and 63.1%, respectively, across participants. Though the overall classification result achieved for the MS vs. NC classification problem was lower than that achieved for MA vs. NC, it is important to note that large inter-participant variation was observed for the former task. High accuracies for the MS vs. NC problem, close to or even exceeding the corresponding MA vs. NC results, were achieved for three of seven participants (> 70%). For one other participant, maximum accuracy achieved for MS vs. NC (63%) was lower than for MA vs. NC, but still significantly above chance. The results for the remaining three participants, however, were all below chance for the MS vs. NC problem. We believe that the greater inter-participant variability was due to the more subjective nature of the mental singing task, in which participants mentally rehearsed self-selected musical pieces and were instructed to try to feel the emotion elicited by the song (it has been suggested that incorporating this self-monitoring element in an emotional induction task can result in an increase in the prefrontal hemodynamic response as compared to more passive emotional tasks [31]). Some individuals may have been able to do this more consistently/effectively than others. It is possible that, if given neurofeedback of their response, even those participants for whom mental singing was ineffective in this study could learn to evoke a detectable response. Collectively, these results are encouraging, and demonstrate the potential of a system-paced NIRS-BCI with one IC state corresponding to either mental arithmetic or mental singing, but suggest that mental singing may not represent a suitable IC state for all users.

In the present study, we wish to expand on our previous results and investigate the feasibility of a system-paced NIRS-BCI with two IC states corresponding to mental arithmetic and mental singing. This is desirable because increasing the number of states recognized by the system increases the functionality/information transfer rate of the BCI. For example, a system-paced BCI with one IC state recognizes two different states (i.e., one IC state and the NC state) and thus allows for a two-choice system (e.g., IC = "yes' and NC = "no"). The addition of a second IC state increases the number of recognized states to three (i.e., the two IC states and the NC state). This in turn allows for a three-choice system (e.g., IC_a _= "yes", IC_b _= "no" and NC = "choosing not to respond"). By increasing the number of recognized states, one increases the number of distinct messages the user can convey.

This is the first NIRS-BCI study to attempt single-trial classification of more than two intentionally- and autonomously-generated mental states (i.e., not dependent on external prompting, and thus suitable for active BCI control). More specifically, this is the first attempt at distinguishing two IC states corresponding to two different cognitive tasks - mental arithmetic and mental singing - and an explicit NC state. We expect to achieve promising classification results in the cases for which both MA and MS are individually distinguishable from the NC state [14].

## Methods

### Participants

Seven able-bodied adults (two male, mean age = 25.7 ± 3.1 years) were recruited from the students and staff at Holland Bloorview Kids Rehabilitation Hospital (Toronto, Canada). Individuals were excluded from participation if they had any condition that could adversely affect either the measurements or their ability to follow the experimental protocol. Ethical approval was obtained from Holland Bloorview Kids Rehabilitation Hospital and the University of Toronto. All participants provided signed consent.

### Instrumentation

**Figure 1:**
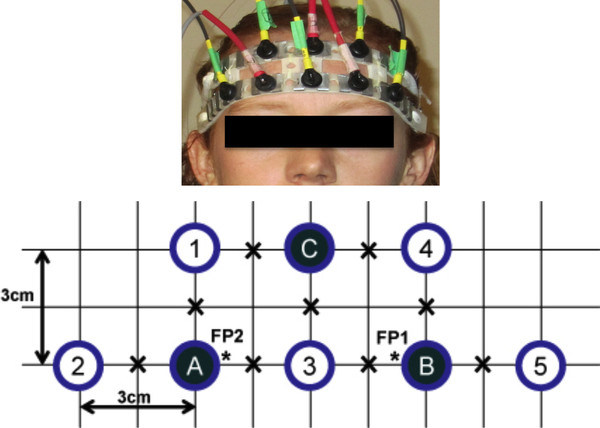
**Source-detector configuration**. Source-detector configuration. Each open circle represents a source-pair comprising one 690 nm and one 830 nm source fibre, while each solid circle represents a detector. Only the source-pair/detector combinations with a separation of 3 cm were considered. "X" denotes a point of interrogation. "*" denotes the approximate FP1 and FP2 positions of the International 10-20 System.

Signals were acquired using a multichannel frequency-domain NIRS instrument (Imagent Functional Brain Imaging System from ISS Inc., Champaign, IL). Ten NIR sources and three photomultiplier tube detectors were secured against the participant's forehead using a flexible headband, as shown in Figure 1. The ten sources were grouped into five pairs, each containing one 690 nm and one 830 nm source, so that each location could be probed by the two wavelengths concurrently. The headband was placed on the participant's forehead such that the bottom row of optodes sat just above the eyebrows, and the center column of optodes was in line with the nose. Nine locations within a 27 cm^2 ^trapezoidal area were probed, as shown in Figure 1. In the given configuration, we considered only signals arising from source-detector pairs (henceforth referred to as "channels") with a separation of 3 cm, which is generally accepted to be the ideal source-detector separation for measuring cortical haemodynamics [32]. This yielded a total of 18 channels (i.e., 3 detectors × 3 source-pairs per detector × 2 wavelengths per source-pair). Data were sampled at 31.25 Hz.

Positioning the headband to achieve adequate coupling of the optodes to a participant's forehead generally took approximately 5-10 min.

### Intentional control states - mental arithmetic and music imagery

For the mental singing task, participants silently rehearsed self-selected musical pieces that they felt would elicit within them a strong, positive emotional response. They were instructed to make an effort to feel the emotion of the song, rather than just passively recite the lyrics or tune.

For the mental arithmetic task, participants performed a sequence of simple mathematical calculations beginning with the subtraction of a small number (between four and thirteen) from a three digit number, and continued throughout the task interval with successive subtractions of the small number from the result of the previous subtraction (e.g., 753-13 = 740, 740-13 = 727, 727-13 = 714, etc.). The calculation the participant was to perform during a given system-vigilant period was displayed on the screen. A different calculation was given for each system-vigilant period of a given session.

### Experimental protocol

**Figure 2:**
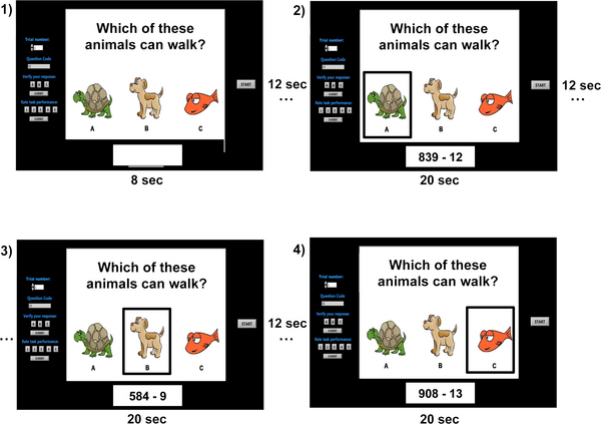
**Example trial stimulus sequence and timing**. Example trial stimulus sequence and timing diagram. In this example, the participant would enter the IC state during intervals 2) and 3) - to select responses A and B - and would remain in the NC state at all other times. The task cue at the bottom of the display indicates that this is a mental arithmetic trial. Note that at the end of each trial, when participants were asked to explicitly verify which answer(s) they selected, they also gave a rating, on a scale of 1-5, of how engaged they felt they were in the task during the trial. These data were used for verification purposes only and were not used in the quantitative analysis.

Each participant completed three experimental sessions which were conducted on different days. During each session, participants performed a total of 32 trials. In each trial participants were visually presented with a question and three possible responses. The three choices were highlighted in sequence for periods of 20 s each. These 20 s periods constituted the system-vigilant periods of the system-paced paradigm, and were separated by 12 s intervals (to allow hemodynamics to return to the no-control/baseline state after activation). The timing of an example trial is shown in Figure 2. Within a given session, no question was repeated. The same 32 questions were used in the three different sessions, but the order was randomized for each.

Participants were instructed to answer the questions by eliciting the indicated IC state (i.e., MA or MS) throughout the intervals in which their desired response(s) were highlighted. There was not necessarily a single correct answer; there could be one, two, three or no correct answers. During the intervals in which they did not wish to make a selection, participants were not required to control their mental activity in any particular way, but rather were told to allow natural thought patterns to occur without restriction. This represented the "no-control" state. To ensure that the data during the system-vigilant periods could be properly labeled as MA, MS or NC, we included only questions with obvious answers (see example in Figure 2) and participants were asked to explicitly verify their selection(s) at the end of each trial. NIRS data were not recorded during this verification period. The protocol was designed such that 72 MA, 72 MS, and 144 NC periods were recorded across the three sessions.

Note that this multiple choice question paradigm is not the ideal application for a system-paced NIRS-BCI with two IC states (i.e., a three-state system). This protocol was designed primarily to facilitate investigation of a system-paced BCI with one IC state (i.e., a two-state system) [14], thus the selected application reflects this. A more suitable application for a three-state system would be one in which the participant could select one of three (rather than one of two) different options during each system-vigilant period; for example, yes/no questions with the possible choices of "yes", "no" and "choosing not to respond". However, since the objective in this work is simply to determine if the three mental states can be automatically differentiated from one another on a single-trial basis, the use of these data is justified.

### NIRS data pre-processing

For each trial, each of the 18 signals (i.e., 2 wavelengths at 9 interrogation locations) was first normalized by its own mean and standard deviation in order to account for inter-trial differences in sensor coupling due to removal of the headband between and (at the participant's request) within sessions. The signals were also linearly detrended to mitigate any effects of instrumentation-related drift. The 20 s system-vigilant periods, of which there were three per trial, were then extracted and grouped into MA, MS and NC samples.

The raw normalised light intensity signals for each system-vigilant period were low-pass filtered in order to mitigate physiological noise due, primarily, to respiration (0.2-0.3 Hz) [33], cardiac activity (0.8-1.2 Hz) and the Mayer wave (approximately 0.1 Hz) [34]. A 3rd-order Chebyshev type II filter was designed with cut-off frequency at 0.1 Hz, stop frequency at 0.5 Hz, pass-band loss of no more than 6 dB, and at least 50 dB of attenuation in the stop-band.

### Feature extraction

Consistent with what we know about the hemodynamic response, we expected to see a change in the amplitude of the light intensity signals after the commencement of the mental task, as the concentrations of oxygenated and deoxygenated haemoglobin change (a result of the haemodynamic response) and in turn alter the absorption properties of the cortical tissue [35]. We found in an earlier study that the slope of the linear regression line fit to the signal within the system-vigilant period was effective for discriminating the intentional control states individually from the no-control state [14]. This result corroborated the findings of other studies which had success classifying mental activity from a controlled rest state using similar amplitude-based features [4,5,36].

As in the earlier study, to capture the unique temporal response for each individual (there could be intersubject variability in time for hemodynamic response to peak, number of peaks, etc.), as well as any temporal differences between the activities, we considered as features the slope of the regression line fit to the signal over multiple time windows within the 20 s response period. Each time interval was defined by a start time and an end time, where start times ranged from 0 to 15 s, and end times ranged from 5 to 20 s, both in 5 s increments. All possible combinations of start and end times, where the latter exceeded the former, were considered as valid time intervals for feature calculation. In total, ten different time windows were considered. Thus the resultant feature pool consisted of 180 candidate features comprising the slope of the regression line fit to each of the 18 channels over each of the 10 time windows.

### Feature selection and classification

In this study, the classification problem of interest is mental arithmetic vs. mental singing vs. the no-control state. A linear discriminant analysis (LDA) classifier was trained on optimal feature subsets selected using a standard genetic algorithm (GA). Such random search algorithms can allow for the evaluation of a search space more efficiently than most other heuristic search methods [37]. The GA parameter values used are listed in Table 1. Feature selection was based on the wrapper method - that is, candidate feature subsets were evaluated for their predictive performance using the learning algorithm of interest [38]. To reduce search time, and to avoid the "curse of dimensionality" (i.e., to maintain an adequate ratio of training sample size to feature subset dimensionality), we explicitly prescribed the subset dimensionality of interest. Based on preliminary analyses, we chose to consider feature subsets with dim = 8, 9, 10, 11 and 12.

**Table 1:** GA parameters

Parameter	Value
Population Size	250

Search space dimensionality	180

Elite count	1

Parent selection	roulette- wheel

Crossover function	scattered

Crossover rate	0.7

Mutation function	Uniform

Mutation rate	0.2

Max generations	30

Fitness function	LDA

Fitness value	mean probability of error

**Figure 3:**
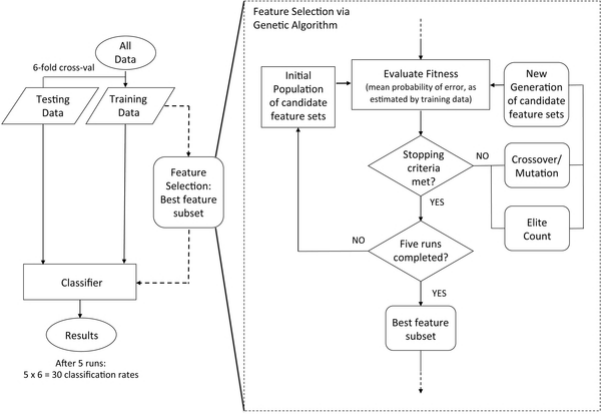
**Classification procedure**. Classification procedure. This procedure was performed on a per-participant basis for each feature subset dimensionality under investigation, specifically dim = 8, 9, 10, 11 and 12.

The classification strategy used in this study is depicted in Figure 3. A six-fold cross-validation was used to estimate the classification accuracy. For each fold of this external cross-validation, five independent runs of the genetic algorithm were performed on the training data. Within the genetic algorithm, LDA served as the fitness function, and the mean probability of error, as estimated by the training set, was selected as the fitness value. Of the five feature sets selected over the five runs of the GA, the set yielding the lowest mean probability of error was used with the training set to train the classifier in the given fold of the external cross-validation. Classification accuracy was then determined for the test set (note that for each fold of the cross-validation, the test set was not involved in either the feature selection or the training of the classifier). A total of five runs of the six-fold cross-validation was performed. Thirty accuracy measures were thus obtained, from which a mean classification accuracy was calculated. Note that adjusted accuracies were used, rather than the standard accuracy measure, to account for bias due to the imbalanced classes (recall there were 144 NC, 72 MA, and 72 MS samples). For a two-class problem, adjusted accuracy (AA) is calculated as [39]

$$\text{AA}=\frac{sensitivity+specificity}{2}$$

Writing this in general terms for a k-class problem gives

$$\text{AA}=\frac{\sum_{j=1}^{k}\left(PC_{j}\right)}{k}$$

where PC_j _is the percentage of correctly classified samples from class *j*. This expression was used to calculate the adjusted accuracies for the MA vs. MS vs. NC classification problem.

To confirm the validity of our classification accuracy estimates, we repeated the classification procedure described above with randomized class labels. If our classification algorithm was properly configured, these results should be at approximately chance level (i.e., 33% for a three-class problem).

## Results

**Figure 4:**
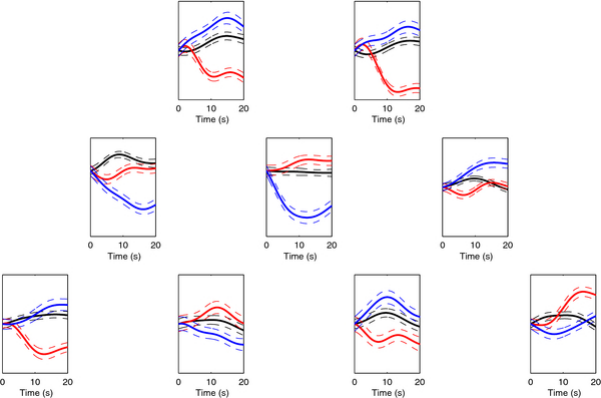
**Example hemodynamic signals for mental arithmetic, music imagery and no-control**. Normalized light intensity versus time plots showing the hemodynamic response for mental arithmetic (red), music imagery (blue) and no-control (black) over the 20 s system-vigilant period. Only the signals from the 830 nm sources are shown. For each task, the signals shown are the average over all samples for one of the participants for whom the three-class classification was successful (P7). Dashed lines indicate standard error.

An LDA classifier trained on a 10-dimensional feature set allowed for the most accurate classification of MA vs. MS vs. NC across participants, yielding an average accuracy of 56.2%. The overall average classification accuracy, as well as all individual participant accuracies, significantly exceeded chance at *α *= 0.01 (note that the upper confidence limit of chance for a three-class problem, *n *= 288 trials and *α *= 0.01 is 40.4% [40]). However, as expected, for three participants (P2, P3 and P4) MS was classified near chance levels, and thus even though the overall accuracy exceeds chance, the MA vs. MS vs. NC classification cannot be considered successful for these participants. Across the four candidate participants (i.e., those participants for whom MA and MS were previously found to be individually differentiable from NC, specifically P1, P5, P6 and P7), an average accuracy of 62.5% was achieved. Further, each of the three classes (i.e., MA, MS and NC) were classified well in excess of chance for all four participants. Figure 4 shows, for one of these participants (P7), the average hemodynamic response for each class over the 20 s system-vigilant period at each of the nine interrogation locations. Note the distinct differences in the response among the three tasks.

Table 2 reports the results for the MA vs. MS vs. NC classification problem (LDA, dim = 10). Along with the overall classification accuracies, it includes the per-class classification accuracies, and the overall classification accuracies for the same data but with randomized class labels. As expected, these values are all approximately 33%.

**Table 2:** MA vs. MS vs. NC classification results: LDA trained on 10-dimensional feature set

Participant Number	Proper Labels				Randomized Labels
	
	AdjustedAccuracy^1^(%)	NC correct (%)	MA correct (%)	MS correct (%)	Adjusted Accuracy (%)
**1**	**64.5 **± **6.9**	**65.6**	**64.3**	**63.6**	32.7 ± 8.1

**2**^2^	49.0 ± 7.8	55.4	55.6	36.1	33.1 ± 6.7

**3**^2^	46.5 ± 8.0	48.1	54.2	37.2	31.8 ± 6.7

**4**^2^	47.9 ± 5.5	51.7	54.7	37.4	32.7 ± 5.2

**5**	**63.6 **± **6.5**	**60.0**	**69.1**	**61.6**	33.4 ± 6.5

**6**	**55.0 **± **6.0**	**54.5**	**63.0**	**47.5**	32.4 ± 5.6

**7**	**66.8 **± **5.8**	**67.6**	**71.4**	**61.3**	33.4 ± 6.0

Mean (all participants):	56.2 ± 8.7	50.4	54.0	43.1	32.8 ± 0.58

**Mean (Participants P1,P5-P7)**^3^	**62.5 **± **5.1**	**61.9**	**67.0**	**58.5**	**33.0 **± **0.54**

^1^The overall average classification accuracy, as well as all individual classification accuracies, are significantly greater than chance (α = 0.01)

^2^As expected for this participant, classification of MS is very near chance level.

^3^Participants for whom both MA vs NC and MS vs NC could be classified with accuracy significantly exceeding chance [16]. These participants were considered candidates for the MA vs MS vs NC classification problem.

## Discussion

The results achieved for the four candidate participants are very encouraging. Accuracies for three of these participants approached 70%, the level cited by some as being necessary for effective BCI communication [41]. With training/practice, these individuals could potentially meet and exceed this level [42]. A three-state system offers a significant increase in functionality/information transfer rate over a two-state system. The findings support the potential of a three-state system-paced NIRS-BCI with intentional control states corresponding to mental arithmetic and mental singing. For three other participants, however, mental singing was ineffective, which suggests that a three-state system based on this task may not be suitable for all users.

A potential source of inter-participant variation in the reported classification accuracies is the inter-individual difference in scalp-cortex distance over the sinus frontalis. Specifically, as sinus volume increases, NIR light traverses a decreasing volume of grey matter, resulting in diminished sensitivity of the measurement to cortical activity [43].

To the best of our knowledge, only two other NIRS-BCI studies have attempted single-trial classification of greater than two mental states [44,45]. However, these studies focused on passive BCI applications [46] (specifically, for enhancing human-computer interaction in gaming systems) and used complex tasks that depend on external cues/stimuli and are therefore unsuitable for an active BCI in which the user should be able to autonomously and spontaneously perform the task (note that though we used visual cues in the MA task in order to keep the experiment controlled, the cues would not be necessary in a practical system; the user could easily select the initial calculation to perform independently). Specifically, they classified rest and two different difficulty levels of externally-cued spatial tasks (76.7% accuracy) [45] or computer game play (54% accuracy) [44]. The higher accuracies reported in [45] as compared to our results could be attributed to the following two differences in the studies: 1) the spatial task used in [45] was more complex than either the mental arithmetic or mental singing task, and could have resulted in greater activation that was more clearly distinguishable from rest; and 2) in our study we differentiated the two tasks and a no-control state, where the participant's brain activity was unconstrained. In the spatial task study, they distinguished tasks of different difficulties from a "controlled rest" exercise, though they do not explicitly define this rest state. It is possible that brain activity was constrained during this period, allowing for greater discriminability compared to the diverse no-control state used in our study. Also, the spatial task study does not report per-class classification rates, thus it is not clear if all three classes were classified successfully.

## Conclusions

This is the first NIRS study to explicitly investigate the automatic discrimination of three intentionally- and autonomously-generated mental states suitable for active BCI control. Specifically, we classified intentional activity due to the performance of two different cognitive tasks - mental arithmetic and mental singing - and the no-control state, where the user's mental activity is unconstrained. With a ten-dimensional feature set and a linear classifier, an overall classification accuracy of 62.5% was achieved across four candidate participants for the MA vs. MS vs. NC classification problem. All participants attained accuracies well in excess of chance, three of which approached 70%, the level cited by some as being necessary for effective communication [41]. Overall, these results are encouraging and demonstrate the potential of a three-state system-paced NIRS-BCI with two IC states corresponding to mental arithmetic and mental singing.

## Endnotes

^a^The term "no-control" state refers to the natural state existing when the user is not consciously modulating his/her brain activity for the purpose of controlling the BCI output, e.g. during periods of thinking, composing, monitoring or daydreaming [13,47,48].

^b^The upper confidence limit around the theoretical chance level of p = 50% for a two-class problem, given *n *= 144 trials and *α *= 0.01, is 60.6% [40]; thus any classification accuracy above this value can be said to be significantly greater than chance at a confidence level of 99%.

^c^During each session, four different questions appeared for each of the eight possible combinations of the three choices (i.e., neither A, B nor C; A only; B only; C only; A and B; A and C; etc.). One set of the eight possible response combinations yields 12 IC and 12 NC periods. Therefore, (4 sets of eight possible response combinations) × (12 IC periods and 12 NC periods) × (3 sessions) = 144 IC periods and 144 NC periods. The 144 IC periods were split evenly between MA and MS, yielding 72 MA and 72 MS periods.

## Competing interests

The authors declare that they have no competing interests.

## Authors' contributions

SDP designed the study, carried out the data collection and analysis, and drafted the manuscript. AK assisted with data analysis and manuscript revision. TC assisted with study design, data analysis and manuscript revision. All authors read and approved the final manuscript.
